# Supplementary material for: Screening of Breast Cancer from Sweat Samples Analyzed by 2-Dimensional Gas Chromatography-Mass Spectrometry: A Preliminary Study
Source: Cancers (Basel). 2023 May 26;15(11):2939. doi: 10.3390/cancers15112939 (PMC10252040; doi:10.3390/cancers15112939)
Supplement: Supplementary file 1 [file cancers-15-02939-s001.zip › cancers-2370162-supplementary figures.pdf]

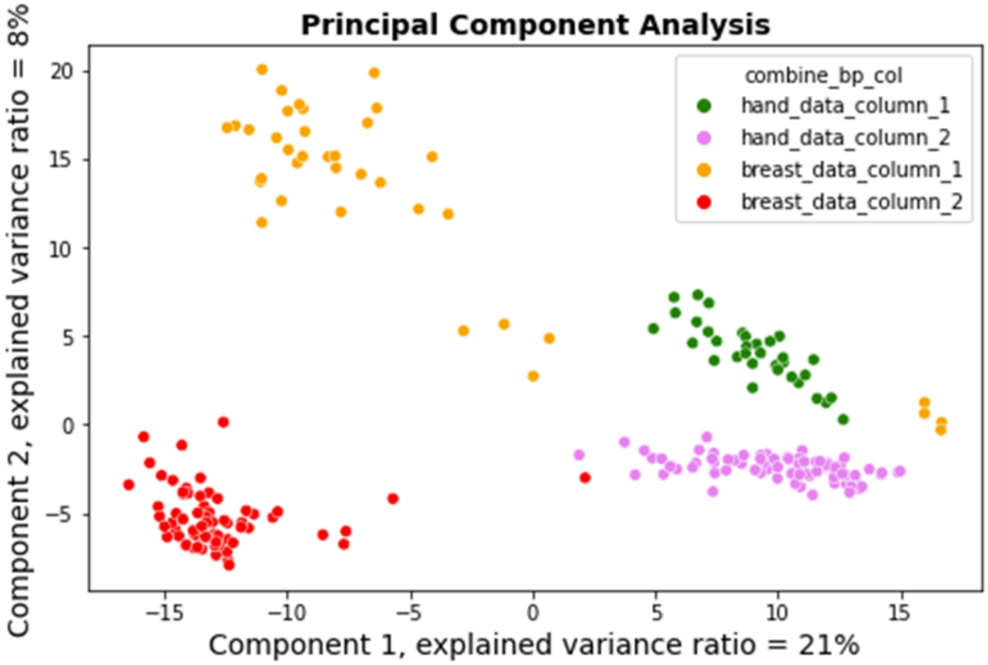

Supplementary Figure S1 – Principal Component Analysis on sweat from hand and breast area collected on BC patients and analysed with different columns. *Green and orange points correspond to hand and breast VOC data respectively analysed with a first GC column. Pink and red dots represent hand and breast VOC data respectively analysed with a second GC column.*

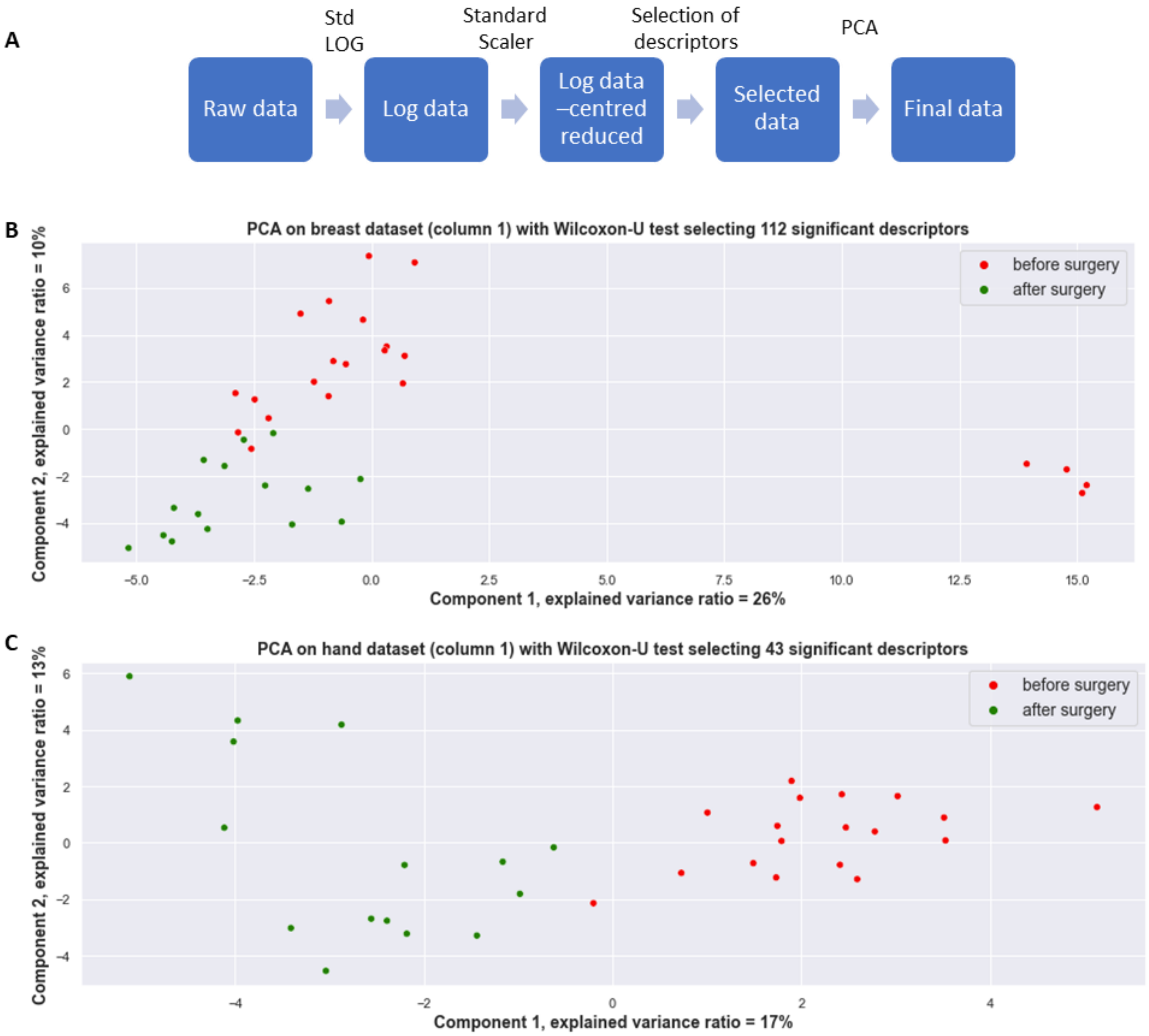

Supplementary Figure S2 – Principal component analysis of VOCs collected on breast and hand area (GC column 1). (**a**) Employed pipeline for PCA construction. (**b**) PCA on the breast dataset with Mann-Witney U descriptor selection for column 1. (**c**) PCA on the hand dataset with Mann-Witney U descriptor selection for column 1.

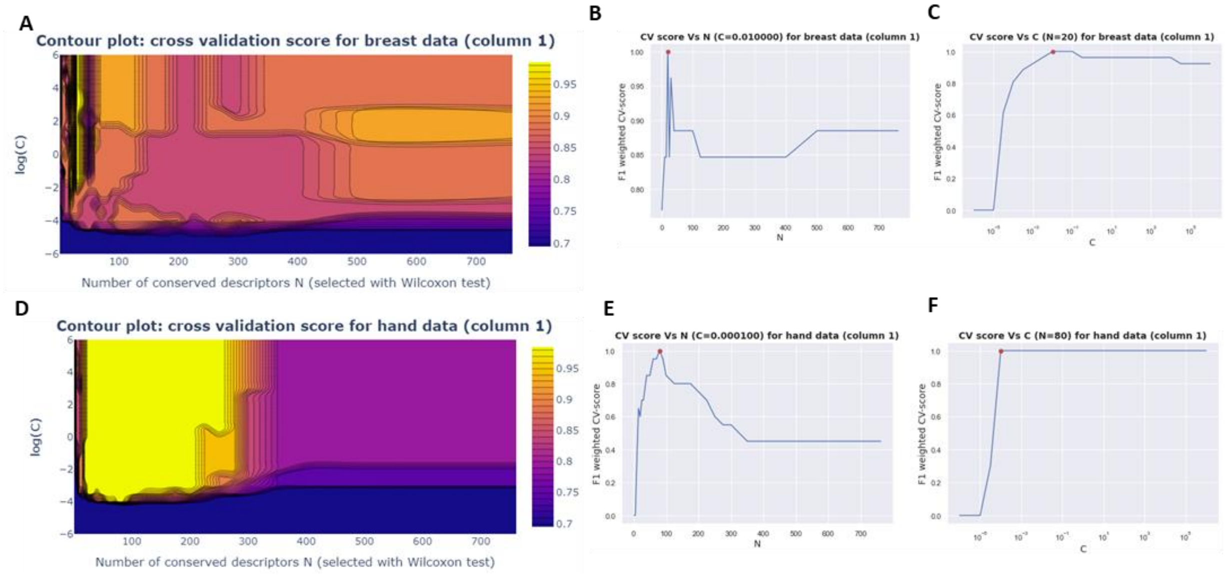

***Supplementary Figure S*3 – Grid search on logistic regression on breast and hand VOC dataset (GC column 1).** (a) contour plot for the breast dataset. (**b**), (**c**). The best model with the lowest complexity is obtained for N=20 and C=0.01, with a CV-score of 1. (**d**) The contour plot for the hand dataset. (**e**) , (**f**) For the COVs collected on the hands, the best model with the lowest complexity is obtained with N= 80 and C = 0.0001.

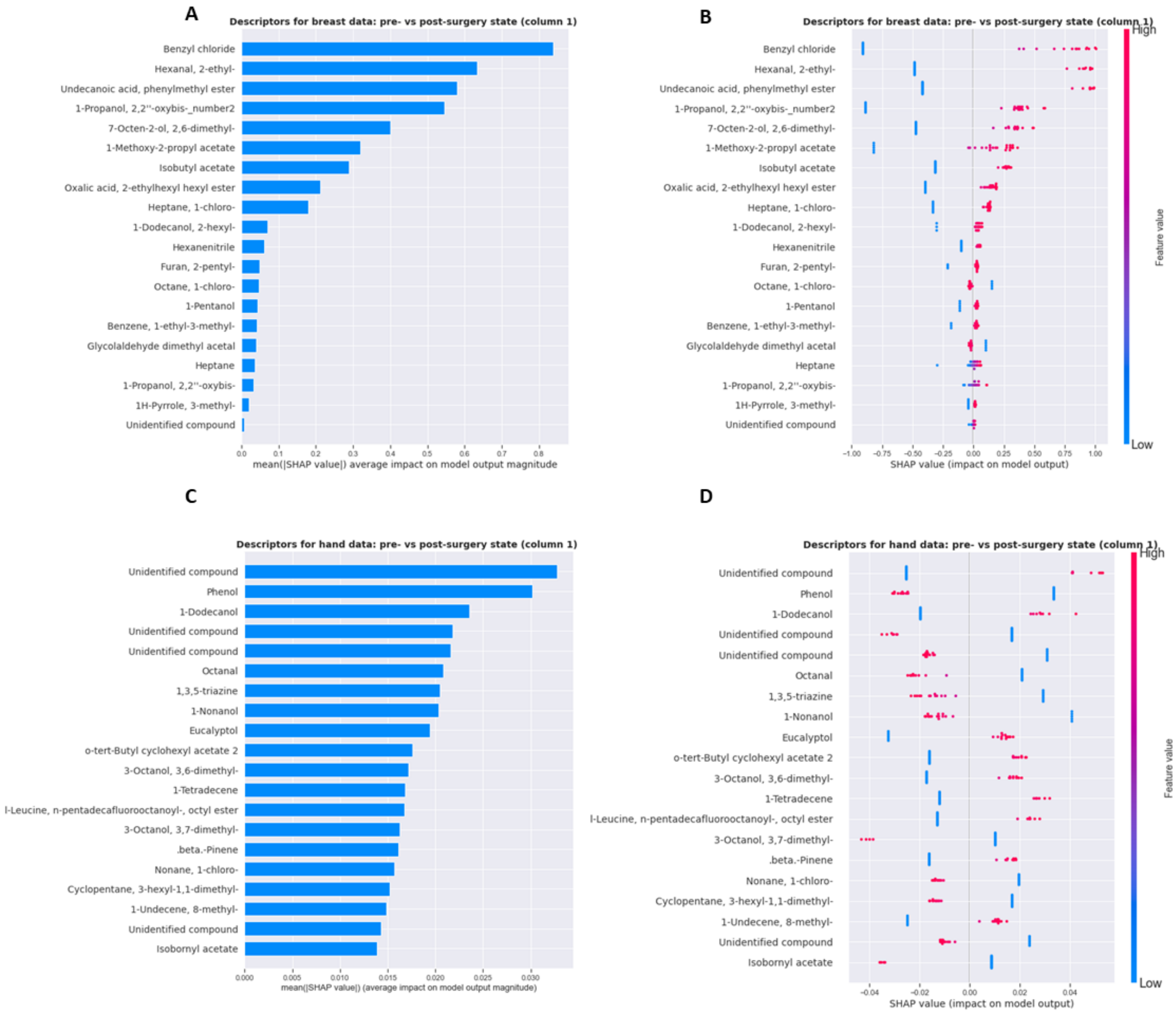

Supplementary Figure S4 – SHAP analysis. *SHAP summary plot for the (**a**) breast data (GC column 1) and (c) hand data (GC column 1). (**b**) Detailed summary plot (SHAP value: negative= " pre-surgery", positive="post-surgery") for the COVs collected on the breast area and (**d**) hand area.*

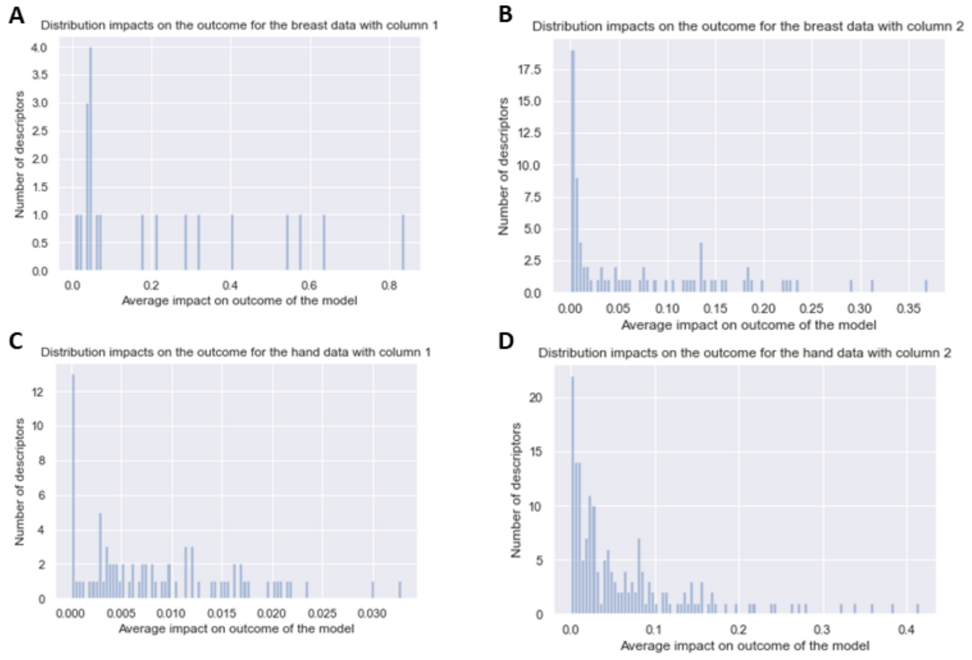

*Supplementary Figure S5 - Distribution impact of the descriptors following SHAP analysis on the outcome for the breast data with GC column 1 (**a**) and GC column 2 (**b**) and the hand data for column 1 (**c**) and column 2 (**d**).*

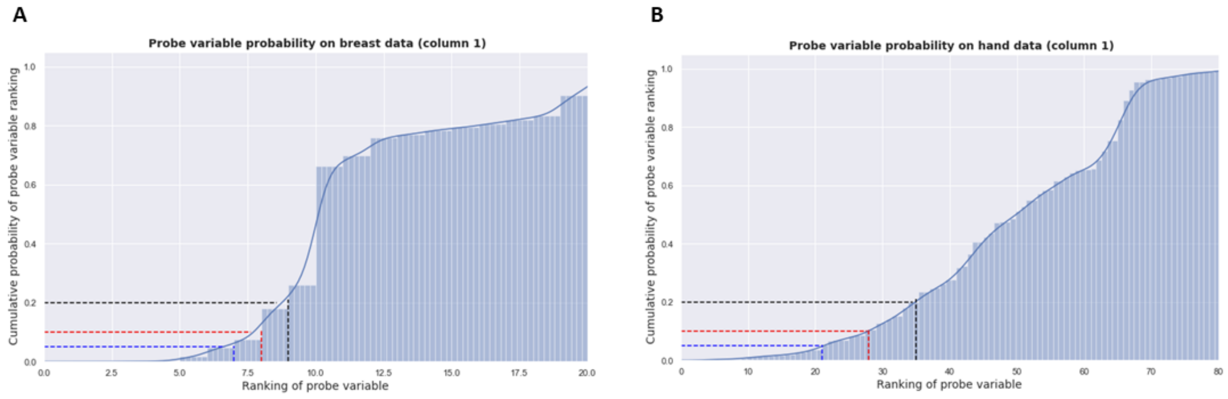

Supplementary Figure S6 – Probe variable method for (a) the breast data and (b) the hand dataset on VOCs analysis conducted with GC column 1.

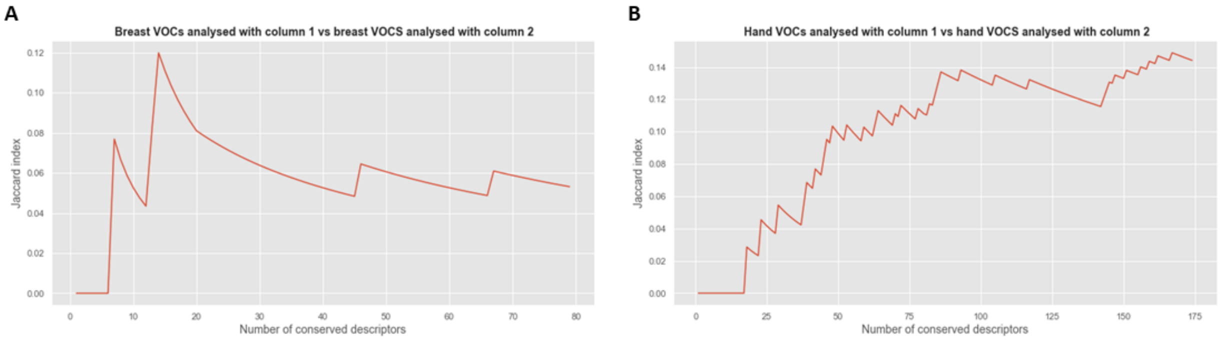

Supplementary Figure S7 - Jaccard index of the retained descriptors between (a) breast collected VOCs analysed with GC column 1 vs analysed with GC column *and (**b**) hand-collected VOCs analysed with GC column 1 vs analysed with GC column 2.*
